# C-type lectin-like receptor (CLEC)-2, the ligand of podoplanin, induces morphological changes in podocytes

**DOI:** 10.1038/s41598-022-26456-9

**Published:** 2022-12-26

**Authors:** Keiko Tanaka, Masafumi Tanaka, Nobuo Watanabe, Masatoshi Ito, Ira Pastan, Masahiro Koizumi, Taiji Matsusaka

**Affiliations:** 1 Departments of Basic Medicine, Tokai University School of Medicine, Isehara, Japan; 2 Molecular Life Science, Tokai University School of Medicine, Isehara, Japan; 3 Emergency and Critical Care Medicine, Tokai University School of Medicine, Isehara, Japan; 4 Internal Medicine, Tokai University School of Medicine, Isehara, Japan; 5 Support Center for Medical Research and Education, Tokai University School of Medicine, Isehara, Japan; 6 Division of Kidney, Diabetes and Endocrine Diseases, Okayama University Hospital, Okayama, Japan; 7 Laboratory of Molecular Biology, Center for Cancer Research, National Cancer Institute, NIH, Bethesda, MD USA

**Keywords:** Kidney diseases, Mechanisms of disease

## Abstract

Podoplanin (PDPN) is intensely expressed on the podocyte membrane in an evolutionally conserved manner. CLEC-2, the endogenous ligand of PDPN, is highly expressed in platelets and also exists in a soluble form in plasma. Normally, podocytes are sequestered from CLEC-2, but when the glomerular barrier is injured, podocytes gain access to CLEC-2. We tested the effects of CLEC-2 in podocytes in vitro and in vivo*.* Cultured podocytes treated with Fc-CLEC-2 demonstrated that CLEC-2 induced the dephosphorylation of ezrin, radixin, and moesin (ERM) proteins. Podocytes treated with Fc-CLEC-2 also showed the dissociation of F-actin filaments from PDPN, F-actin degradation, detachment, and round morphology. Next, we perfused normal mouse kidney in vivo with FLAG-CLEC-2. CLEC-2 induced dephosphorylation of ERM and widening of the foot processes of podocytes. Platelets were detected by immunostaining for CD41 in the urine of mice with podocyte injury, indicating that podocytes can encounter platelets when glomeruli are injured. Collectively, these observations suggest that when platelets leak through the injured glomeruli, CLEC-2 from the platelets acts on PDPN in podocytes and induces morphological change and detachment, which may further aggravate podocyte injury. Thus, PDPN on podocytes may work as a leaked-platelet sensor.

## Introduction

Podoplanin (PDPN) is a membranous mucin-type *O*-glycosylated glycoprotein, which is negatively charged by abundant sialic acid. PDPN is expressed on the surface of various types of cells, including kidney podocytes, alveolar epithelial cells, lymphatic endothelial cells, stromal fibroblastic reticular cells (FRCs) of lymph nodes^1^, and tumors^2^. Among these, PDPN is most intensely expressed on podocytes and its expression is evolutionally conserved^3^.

PDPN was found to be the endogenous ligand of C-type lectin-like receptor 2 (CLEC-2) on platelets and is involved in platelet aggregation induced by tumor cells^4^, which facilitates invasion and metastasis of the tumor^2,5,6^. During developmental stages, CLEC-2 in platelets can interact with PDPN in lymphatic endothelial cells, and activated platelets facilitate blood-lymphatics vessel separation^7^. Platelet activation by PDPN also plays a critical role in the differentiation of alveolar duct myofibroblasts^8^.

CLEC-2–PDPN interaction mediates bidirectional signaling. PDPN works as a ligand for CLEC-2 as shown above. In addition, binding PDPN with CLEC-2 influences PDPN-expressing cells. Binding PDPN with CLEC-2 attenuates actomyosin contractility in FRCs in the lymph node^9^, inhibits migration of lymphatic endothelial cells^10,11^, stimulates CCL5 secretion in FRC-like cells in bone marrow^12^, stimulates IGF-1 secretion from stromal cells^13^, and attenuates inflammatory responses in a subset of Th17 cells^14^.

The role of PDPN in podocytes is not fully elucidated. Decreased expression of PDPN in podocytes is associated with foot process effacement, proteinuria, and decreased glomerular selective permeability in several animal models^15–17^. In biopsy specimens of patients with minimal change nephrotic syndrome, PDPN staining was decreased in the proteinuric state, and recovered when proteinuria was normalized^18^. These facts suggest that PDPN is important for maintenance of the normal function of podocytes. The cytoplasmic tail of PDPN interacts with ezrin, radixin, and moesin (ERM) proteins, which bind the actin cytoskeleton and regulate cell shape. It was reported that whole-body *Pdpn*-gene-disrupted mice showed no abnormal renal phenotypes^19^. Podocalyxin-NHERF2 complex and nephrin-ephrin-B1-NHERF2 complexes can bind and maintain ezrin-F-Actin complex^20,21^. This may be a reason for the lack of abnormal phenotype in podocytes of congenital Pdpn-deficient mice. However, suppression of PDPN by siRNA in cultured podocytes changed cell morphology from an elongated to a round shape along with a change in ezrin distribution^18^.

Normally, podocytes are sequestered from platelets, but when the glomerular barrier is injured, podocytes gain access to CLEC-2 on platelets. In addition, a soluble form of CLEC-2 with molecular weight 25 kDa exists in human plasma^22^. We speculated that CLEC-2, the known ligand for PDPN, may have some biological impact on podocytes. This hypothesis is supported by previous reports that injection of antibodies against specific epitopes of PDPN caused transient proteinuria and foot process effacement in rats^23^. In the present study, we examined the effect of CLEC-2 in podocytes in vitro and in vivo.

## Results

### Effects of CLEC-2 on in vitro podocytes

**Figure 1:**
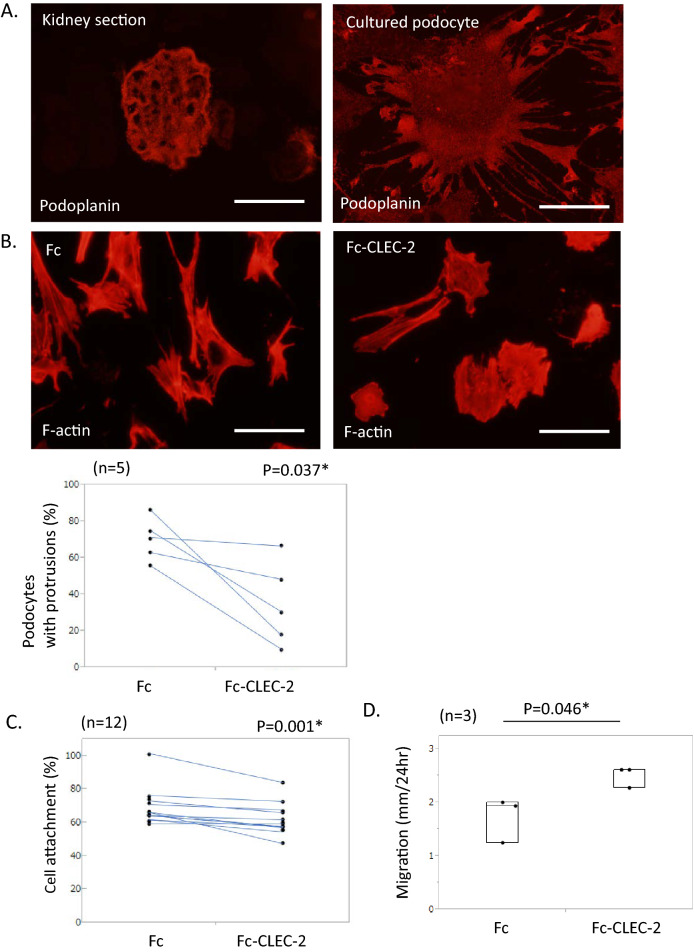

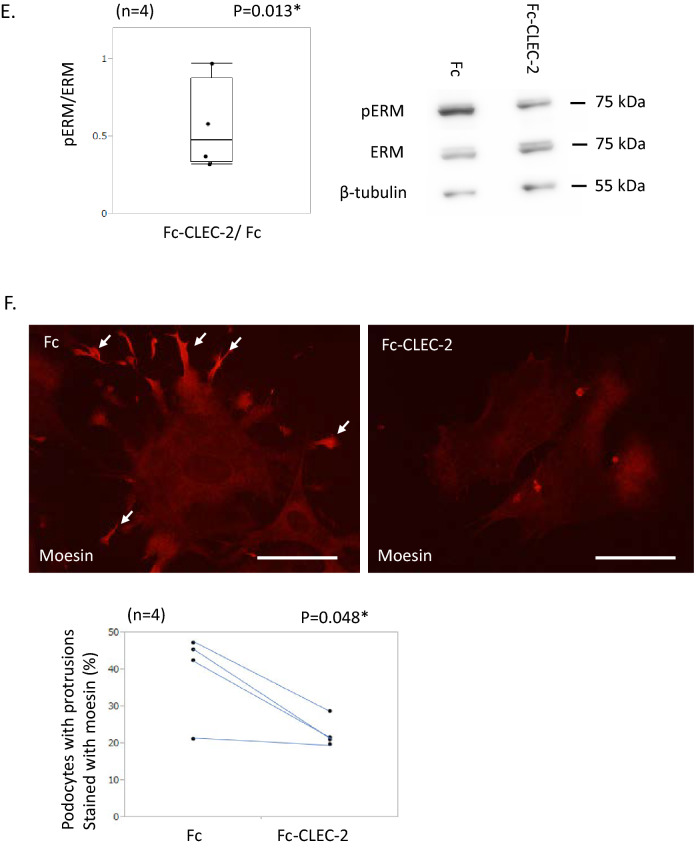
In vitro study with Fc and Fc-CLEC-2. (**A**) Representative images of kidney sections and cultured podocytes, stained with podoplanin (PDPN). PDPN is intensely stained both in in vivo podocytes and cultured podocytes. Scale bar: 50 μm. (**B**) Representative images of cultured podocytes, stained with phalloidin after 1 h incubation at 37 °C with Fc or Fc-CLEC-2. Podocytes incubated with Fc-CLEC-2 showed a round shape with degradation of F-actin, while those with Fc showed an elongated morphology with numerous F-actin filaments. Scale bar: 100 μm. Podocytes with protrusions were significantly reduced by Fc-CLEC-2. (**C**) Adhesion assay. The percentage of attached podocytes was evaluated after 1 h incubation at 37 °C with Fc or Fc-CLEC-2. Podocytes incubated with Fc-CLEC-2 showed less attachment than those with Fc. (**D**) Migration assay. The distance of migration of podocytes was measured while incubated at 37 °C with Fc or Fc-CLEC-2 for 24 h. Podocytes incubated with Fc-CLEC-2 showed greater migration than those with Fc. (**E**). Western blot analysis for ERM and pERM after treatment of Fc or Fc-CLEC-2 at 37 °C for 1 h. The pERM/ERM ratio was 0.47-fold decreased in podocytes with Fc-CLEC-2, compared to those with Fc. The images of full-length blots are shown in Supplementary Figs. 2 and 3. Ezrin: 81 kDa, Moesin: 75 kDa, β-tubulin 55 kDa. (**F**) Representative images of cultured podocytes, stained with moesin after 1 h incubation at 37 °C with Fc or Fc-CLEC-2. Fc increased the number of protrusions that were positive for moesin staining, and Fc-CLEC-2 markedly decreased them. Scale bar: 50 μm. The podocyte shape was recognized by phase contrast imaging (shown in supplementary Fig. 5).

We first tested the effects of recombinant Fc-human CLEC-2 on cultured mouse podocytes. Although most proteins characteristic to podocytes, such as nephrin and podocin, are rapidly downregulated upon in vitro culture, PDPN staining was maintained in primary cultured podocytes with similar intensity to in vivo podocytes (Fig. 1A). As reported previously^3^, a pull-down experiment showed that Fc-human CLEC-2 bound to mouse PDPN (S.Fig. 1A). The majority of podocytes treated with Fc protein for 1 h showed an elongated morphology with sharp protrusions and had numerous F-actin filaments. In contrast, podocytes treated with Fc-CLEC-2 showed a round cell shape without sharp protrusions and a decrease in F-Actin (Fig. 1B). Furthermore, podocytes treated with Fc-CLEC-2 showed less adhesion to the collagen-1-coated plate within 1 h than the Fc control cells (59.6% vs. 65.3%) (Fig. 1C). Podocytes with Fc-CLEC-2 showed more migration than the Fc control (2.62 vs. 1.93 mm/24 h) (Fig. 1D).

**Figure 2:**
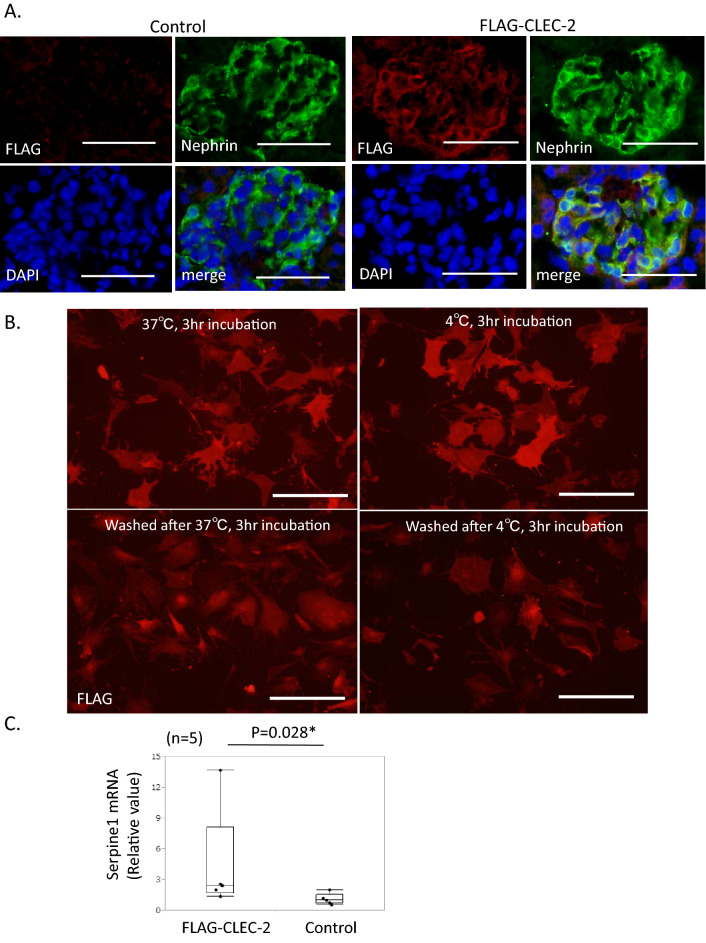

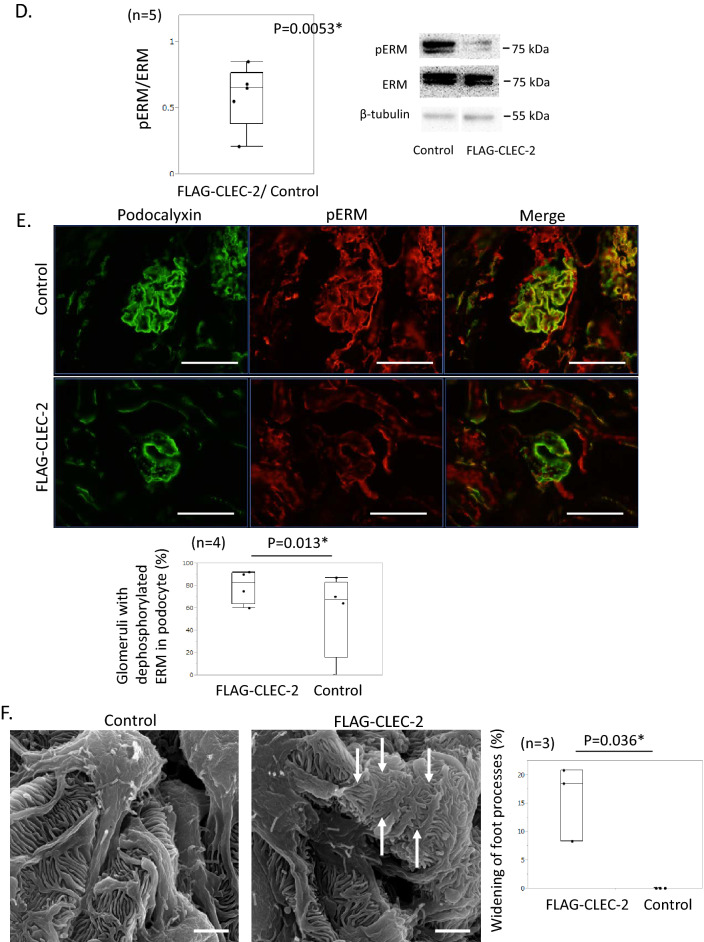
In vivo study with FLAG-CLEC-2 protein. (**A**) Double immunostaining for FLAG and nephrin in kidneys with or without infusion of FLAG-CLEC-2. After infusion of FLAG-CLEC-2, glomeruli were intensely stained for FLAG in nephrin-positive podocytes. Scale bar: 50 μm. (**B**) Internalization assay using cultured podocytes. Representative images of podocytes stained for FLAG, after 3 h incubation at 37 °C or 4 °C with or without subsequent washing with a stripping buffer. At either temperature, FLAG staining diminished after washing, indicating that FLAG-CLEC-2 was not internalized within the cells. Scale bar: 200 μm. (**C**) Serpine1 mRNA in the glomeruli (relative amount). Serpine1 mRNA expression was 2.45-fold increased in the mice with FLAG-CLEC-2 infusion. (**D**) Western blot analysis of glomerular lysate for ERM. Phosphorylation of ERM proteins was 0.65-fold decreased by FLAG-CLEC-2. The images of full-length blots are shown in Supplementary Figs. 7 and 8. Ezrin: 81 kDa, Moesin: 75 kDa, β-tubulin 55 kDa. (**E**) Dephosphorylation of ERM in podocytes by FLAG-CLEC-2 perfusion. Double immunostaining showed an intense signal for pERM (red) in podocytes labeled by podocalyxin staining (green) in control mice (upper panels). In the mice perfused with FLAG-CLEC-2, some podocytes lack pERM staining. Scale bar: 50 μm. (**F**) SEM images of the foot processes. Podocytes perfused with FLAG-CLEC-2 exhibited widening of foot processes (arrows) in 18.5% of visual fields. Original magnification: × 8000. Scale bar: 2 μm.

**Figure 3:**
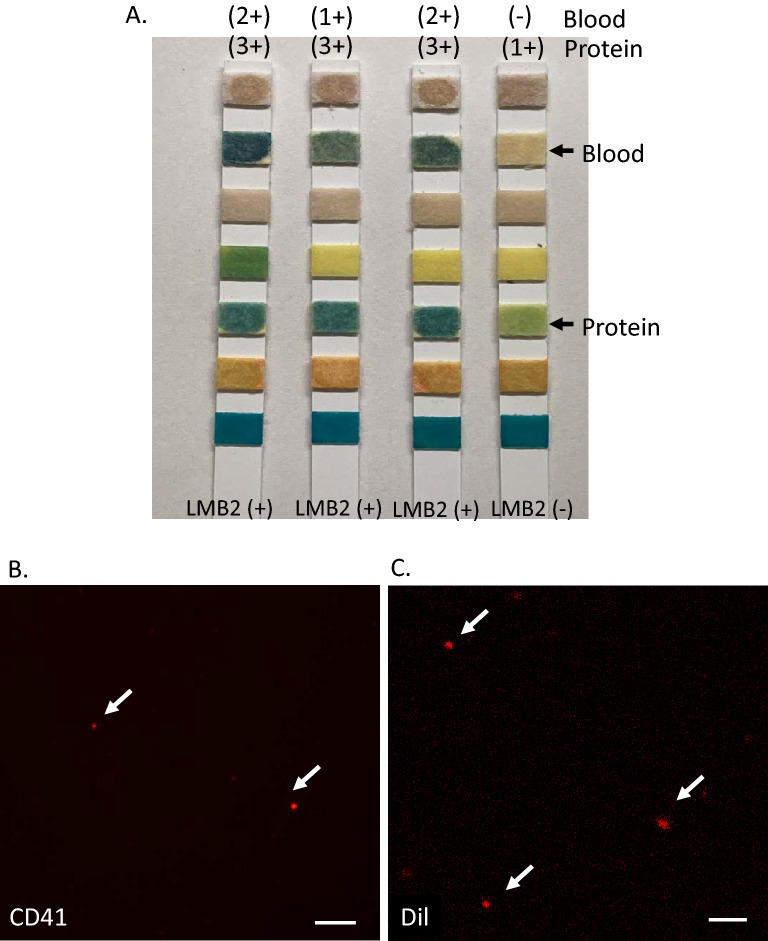
Detection of urinary platelets in podocyte injured mice. (**A**) Urinary protein and occult blood were detected by urine test strip in the urine 5 days after LMB2 injection [LMB2 (+)]. Those reactions were negative for LMB2-untreated mice [LMB2(−)]. Blood changes white paper to green, and protein changes yellow paper to green. Note that normal mice show 1 + proteinuria due to high concentration of small molecular weight proteins. (**B**) Confocal microscopy of Cy3-CD41 platelets in the urine. Immunostaining for CD41 showed platelets (arrows) were detected in the urine 5 days after LMB2 injection. Scale bar: 20 μm. (**C**) Confocal microscopy of DiI-platelets in the urine. DiI labeled platelets (arrows) were detected in the urine of mice injected with DiI platelets 5 days after LMB2 injection. Scale bar: 20 μm. Positive and negative control images for (**B**) and (**C**) are shown in supplementary Fig. 9.

We next studied the phosphorylation status of ERM, which links PDPN with F-actin, to reveal the intracellular signaling induced by CLEC-2. Western blot analysis revealed that treatment with Fc-CLEC-2 reduced the pERM/ERM ratio in cultured podocytes, indicating that CLEC-2 induced dephosphorylation of ERM proteins (Fig. 1E, S.Figs. 2 and 3). Ezrin has been regarded as the major ERM protein in podocytes^24^. Using Western blot analysis and quantitative PCR, we found that ezrin was downregulated in cultured podocytes (S.Fig. 4A and B). Ezrin was not detected by immunostaining in cultured podocytes. Moesin was intensely stained in the protrusions of the Fc control podocytes, and incubation with Fc-CLEC-2 markedly decreased moesin staining (Fig. 1F, S.Fig. 5). These results collectively indicate that CLEC-2 induced the dephosphorylation of ERM proteins, which caused the dissociation of F-actin filaments from PDPN, F-actin degradation, and cell morphological changes.

### The effects of CLEC-2 on in vivo podocytes

To test the effects of CLEC-2 on in vivo podocytes, we generated a new recombinant CLEC-2 protein with a smaller size, because the above Fc-CLEC-2 forms a tetramer with a size of about 240 kDa, which was not expected to reach podocytes through the normal glomerular barrier. The new mouse CLEC-2 protein, FLAG-CLEC-2, exists as a monomer in solution and the size is 30–35 kDa. FLAG-CLEC-2 bound to cultured podocytes, which was competed with Fc-CLEC-2, but not with Fc, confirming the specificity of the binding (S. Fig. 6).

We infused 5 μg/g body weight of FLAG-CLEC-2 into normal mice and excised the kidney 1 h later. Double immunostaining for FLAG and nephrin, a protein specific to podocytes, confirmed that FLAG-CLEC-2 bound to podocytes (Fig. 2A). However, when glomeruli were isolated from mice 1 h after injection of FLAG-CLEC-2, FLAG was not detected by Western blot in the glomerular lysate. This implies that FLAG-CLEC-2 was not internalized into podocytes and that FLAG-CLEC-2 was detached from glomeruli during the glomerular isolation procedure. To test this possibility, cultured podocytes were incubated with FLAG-CLEC-2 (10 μg/mL) at 37 °C and washed with an acidic buffer. FLAG staining in podocytes decreased after washing. When incubation was performed at 4 °C, FLAG was stained in podocytes with similar intensity to those incubated at 37 °C, and the staining was similarly decreased after washing with an acidic buffer (Fig. 2B). These observations indicate that FLAG-CLEC-2 is not internalized into podocytes.

Quantitative RT-PCR analysis of the glomerular RNA revealed that infusion of FLAG-CLEC-2 increased Serpine1 mRNA, a podocyte injury marker^25^, 2.45 (1.68–8.14)-fold compared to controls (Fig. 2C). The Western blot of glomerular lysate revealed that FLAG-CLEC-2 decreased the pERM/ERM ratio 0.65 (0.38–0.76)-fold (Fig. 2D, S.Figs. 7 and 8), indicating that CLEC-2 induced dephosphorylation of ERM proteins. This was also confirmed by double immunostaining of pERM and podocalyxin (Fig. 2E).

In scanning electron microscopy (SEM) images, widening of foot processes was observed in 18.5% of visual fields in the mice treated with FLAG-CLEC-2, contrasting with no such change in control mice, suggesting that CLEC-2 induced a change in the cytoskeleton in foot processes (Fig. 2F).

These findings indicate that CLEC-2 induced dephosphorylation of ERM and concomitant widening of the foot processes of podocytes.

### Urinary platelets excreted by mice with podocyte injury

We tested the possibility that podocytes can encounter platelets when glomeruli are injured. For this purpose, we induced podocyte injury in NEP25 mice by injecting LMB2. Five days after the injection of LMB2 (5 ng/g body weight), NEP25 mice exhibited both urinary protein and urinary blood (Fig. 3A), indicating disruption of the glomerular barrier. Immunostaining of CD41 revealed that CD41 positive platelets were observed in the urinary sediments at this time point (Fig. 3B), but not in those from LMB2-untreated control mice (S.Fig. 9A). To further verify leakage of platelets, platelets were collected from wild-type mice, labeled with DiI, and injected into NEP25 mice 5 days after the injection of LMB2. DiI-labeled platelets were found in the urine collected from the injected mice (Fig. 3C). Thus, platelets in the bloodstream can make contact with podocytes when the glomerular barrier is injured.

## Discussion

The present study revealed that recombinant CLEC-2, the ligand of PDPN, induced significant morphological change, attenuated adhesion, and promoted migration in cultured podocytes. CLEC-2 causes dephosphorylation of ERM and decomposition of PDPN-ERM-F-actin complex. A previous report indicated that PDPN binds to ezrin, whose phosphorylated form tightly connects with F-actin in podocytes^18^. A similar phenomenon has been reported in the FRCs of lymph nodes. CLEC-2 on dendritic cells acts on the PDPN of FRCs and induces dephosphorylation of ERM, disconnection of ERM from the plasma membrane, and a reduction in actomyosin contractility, which elongates FRCs^26^. In both FRCs and podocytes, binding with CLEC-2 attenuates the basal function of PDPN. Similarly, CLEC-2 inhibits the basal function of PDPN in keratinocytes and lymphatic endothelial cells^11,27^, but in these cases the net effect of CLEC-2 is the inhibition of cell migration, which is opposite to that in podocytes. The reason for the opposite direction is not clear, but differences in cell character may be involved. Podocytes are basically static cells and have unique thick actin bundles. In addition, a recent study showed that the stimulation of PDPN acts downstream of VEGF signaling, but not directly on ERM, in lymphatic endothelial cells^28^.

Transient exposure to recombinant FLAG-CLEC-2 protein in a short period (1 h) induced a significant morphological change in intact in vivo podocytes although the effect was modest compared to those in cultured podocytes. The modest effect may be caused by the monomeric feature of FLAG-CLEC-2. In injured glomeruli, polymeric CLEC-2 on platelets may bind to PDPN on podocytes and exert greater impacts. Moreover, in injured glomeruli, nephrin and podocalyxin are rapidly and remarkably downregulated^29^, which bind to NHERF2 and stabilize ezrin-F-Actin complex in intact podocytes^20,21^. Therefore, CLEC-2 may have a more significant impact on injured podocytes than those of the perfusion study.

CLEC-2 is a membrane-bound protein mainly expressed in platelets. In addition, CLEC-2 exists in the circulation as shed or microparticle-bound forms^30^. It was reported that the mean plasma concentration of these soluble forms of CLEC-2 was 59–100 pg/mL in healthy volunteers and was increased to 260–380 pg/ml in patients with platelet-activating diseases^22^. In some patients with thrombotic microangiopathy or disseminated intravascular coagulation, the concentration of soluble CLEC-2 exceeds 1000 pg/ml^31–33^. Because platelets are retained in inflamed glomerular capillaries^34^, the local concentration of soluble CLEC-2 may be higher in glomerular diseases. Nevertheless, these concentrations do not appear sufficient to induce morphological change in normal podocytes considering the high dose of the recombinant CLEC-2 (5 μg/g body weight) used in the present study.

Platelets are smaller than erythrocytes, therefore they can pass through the damaged glomerular barrier in glomerular diseases with hematuria. In fact, urinary platelets were detected in glomerular diseases^35–38^. We also demonstrated that platelets are excreted into urine in our podocyte injury mouse model. We speculate that platelets may pass through the glomerular barrier and act on PDPN in podocytes in kidney diseases. Although urinary platelets have received almost no attention, they may reflect a distinct disease condition.

Taken together, we propose that PDPN on podocytes works as a sensor of platelet CLEC-2, which is leaked through glomeruli after severe injury. Stimulation by CLEC-2 induces morphological change and detachment of podocytes, which appears to further aggravate podocyte injury. Considering that PDPN on podocytes and CLEC-2 on platelets are evolutionally conserved, this system may have some beneficial effects, such as facilitating the repair process. Further study is necessary to establish the role of PDPN on podocytes.

## Methods

### Animal ethics

All animal experiments were approved by the Animal Experimentation Committee of Tokai University School of Medicine. All animal experiments were performed in accordance with relevant guideline and regulations, and the study is reported in accordance with ARRIVE guidelines (https://arriveguidelines.org).

### Recombinant CLEC-2 proteins

Fc-CLEC-2, a fusion of the Fc tag and C-terminal extracellular domain of human CLEC-2 (51–229), was prepared as previously reported^39^. Fc-CLEC-2 expression plasmid was transiently transfected in HEK293 cells using X-tremeGENE 9 DNA (Roche), and Fc-CLEC-2 protein was purified by protein A affinity chromatography (KANEKA KanCapA, Wako). HEK293 cells were kindly provided by Dr. Takehito Sato at Tokai University and used in a previous study^39^. SDS-PAGE and Coomassie Brilliant Blue (CBB) stain confirmed the expected size of bands (Fc; 30 kDa, Fc-CLEC-2; 60 kDa) (S.Fig. 1B). Pull-down assay confirmed that mouse PDPN can bind Fc-CLEC-2 (S.Fig. 1A).

StepTagII-3FLAG-CLEC-2, a fusion of StrepTactin FLAG double tags and the C-terminal extracellular domain of mouse CLEC-2 (51–229), was generated in HEK293 cells transiently transfected with the expression plasmid using polyethyleneimine Max reagent (Polysciences, Inc.). The supernatant of cell medium was added with biotin blocking solution (Biolock, iba Life Science), and StepTagII-FLAG-CLEC-2 protein (hereafter designated as FLAG-CLEC-2) was purified by a StrepTactin Sepharose column (StrepTrap HP, GE healthcare Life Sciences). Mass spectrometric analysis by the LCMS-IT-TOF (Shimadzu) confirmed that the purified protein contained peptides specific to mouse CLEC-2. SDS-PAGE and CBB stain showed double bands around 30-35 kDa (S.Fig. 1B). The deglycosylation by PNGase F (New England Biolabs) changed the two bands to a single band (S.Fig. 1C). Pull-down assay confirmed that mouse PDPN can bind FLAG-CLEC-2 (S.Fig. 1B).

Blue Native PAGE showed that Fc and Fc-CLEC-2 exist as tetramers, and FLAG-CLEC-2 exists as a monomer in aqueous solution (S.Fig. 1D).

### Western blot analysis

Cells were lysed in a lysis buffer containing 1% Triton X-100, 2 mM CaCl_2_, 0.5 mM PMSF, protease inhibitor cocktail (cOmplete, Roche), and 50 mM Tris/HCl (pH7.4). For analysis of phosphorylated protein, 10 mM NaF, 1 mM Na_3_VO_4_, and 5 mM Na_4_P_2_O_7_ were added. The homogenates were centrifuged at 15,000 rpm to remove the insoluble fraction. Each protein sample was separated by SDS-PAGE and transferred onto a PVDF membrane. The protein-blotted membranes were incubated with 1:1000 diluted primary antibodies overnight at 4 °C and then incubated with HRP-conjugated secondary antibodies for 1 h at room temperature. The density of the positive bands was quantified by image analysis with CS Analyzer 3.0 (ATTO). The following primary antibodies were used: pERM (Cell Signaling, #3726), ERM (Cell Signaling, #3142), and β-tubulin (Cell Signaling, #2128).

The antigens of anti-ERM (#3142) and anti-pERM (#3726) antibodies are commonly shared by ezrin, radixin, and moesin.

### Pull-down assay

Podocyte lysate (1.5 g/L, 50 µL) containing 1% Triton X-100 was mixed with 3 μg of Fc-CLEC-2 or FLAG-CLEC-2 for 1 h at 4 °C, and the samples were incubated with KANEKA KanCapA (Wako) or Strep-Tactin Superflow plus (Qiagen), respectively, for 1 h at 4 °C. After removing the supernatant, the beads were washed three times with cell lysis buffer and then incubated in SDS sample buffer. The supernatants were subjected to SDS-PAGE followed by Western blot with anti-PDPN antibody.

### Isolation of glomeruli and primary podocyte culture

Glomeruli were harvested using the bead method as previously reported^29^. They were cultured on a collagen-1-coated dish for 7 days in DMEM/F12 medium containing 5% FCS and 0.5% ITS-A. Outgrowing cells were detached and passaged after removing residual beads and glomeruli. Cells were used for in vitro experiments 1 to 7 days after the first to third passages. Cultured podocytes were treated with Fc or Fc-CLEC-2 (10 µg/mL).

### Isolation of in vivo podocyte mRNA

We utilized RiboTag mice, which express the ribosomal protein, Rlp22, which is tagged with hemagglutinin (HA) only in cells expressing Cre recombinase^40^. The RiboTag mice were crossed with podocyte-specific Cre-expressing mice (*Nphs1*-Cre mice). Podocyte RNA was purified from podocyte polysomes obtained by immunoprecipitation with anti-HA antibody of glomerular lysate.

### Adhesion assay

Primary cultured podocytes were seeded at a density of 30,000/cm^2^ in collagen-1-coated 96-well plates. After 1 of hour incubation at 37 °C with Fc or Fc-CLEC-2 (10 µg/mL) in 0.5% FCS medium, all wells were washed several times with PBS. The number of attached cells was quantified by Cell Counting Kit-8 (Dojindo Laboratories).

### Migration assay

Primary cultured podocytes were seeded at a density of 30,000/cm^2^ within O-rings on collagen-1-coated dishes and cultured to reach 90–100% confluency. After the O-rings were removed, the cells were allowed to migrate for 24 h at 37 °C in 5% FCS medium with Fc or Fc-CLEC-2 (10 µg/mL). The longest migration length was measured.

### Perfusion of mouse kidney with recombinant CLEC-2 protein

C57BL/6 mice (4–6 months of age, approximately 12-19 g body weight) were used for the experiments. Under anesthesia with pentobarbital (50 mg/kg, i.p.) and buprenorphine (0.05 mg/kg, s.c.), the celiac and superior mesenteric arteries were transiently occluded with clips. The kidneys were perfused with 300 μl of PBS or PBS containing 5 μg/g body weight of FLAG-CLEC-2 through a catheter placed in the abdominal aorta at a distal site and then the clips were removed. After 1 h, kidneys were perfused with PBS, harvested, and analyzed by electron microscopy and immunohistochemistry. In some experiments, glomeruli were collected and used for PCR and Western blot analyses.

### Quantitative RT-PCR

Total RNA was extracted from isolated glomeruli with an RNeasy Plus Mini Kit (Qiagen) according to the manufacturer’s instructions. Single-stranded cDNA was prepared from 100 ng of RNA using TaqMan Reverse Transcription Reagents (ThermoFisher). A TaqMan primer probe set (Thermo Fisher) was used for *Gapdh*. For other genes, the following primers were used: *Serpine1*, 5’-AGGATCGAGGTAAACGAGAGC-3’ and 5’-GCGGGCTGAGATGACAAA-3’; *Moesin*, 5’-TCTTATGCCGTCCAGTCTAAGT-3’ and 5’-GGTCCTTGTTGAGTTTGTGCT -3’; *Ezrin*, 5’-CAATCAACGTCCGGGTGAC-3’ and 5’-GCCAATCGTCTTTACCACCTGA-3’. Relative amounts of mRNA were determined using the delta-delta CT method.

### Immunostaining and F-actin staining

Primary cultured podocytes were seeded at a density of 5000/cm^2^ on glass-based dishes 1–2 days before staining. For PDPN and moesin staining, cells were fixed in acetone. For FLAG* and F-actin staining, cells were fixed in 4% paraformaldehyde (PFA) and permeabilized in 0.1% Triton X-100/PBS. After blocking, they were incubated with Alexa Fluor 594-phalloidin (Invitrogen, diluted at 1/100) for F-actin staining. For double staining of FLAG** and nephrin, frozen kidney sections were fixed in acetone. For double staining of pERM and podocalyxin, kidneys were fixed in trichloroacetic acid (TCA) for 1 h, followed by 4% PFA/PBS for 1 h before preparing frozen blocks with OCT to preserve the phosphorylation of ERM. Information regarding antibodies is shown in Table 1. Can Get Signal solution (TOYOBO) was used for FLAG** and nephrin staining of frozen kidney sections.

**Table 1:** Antibodies for immunostaining.

Antibody	Company	Cat. number	Dilution or concentration
PDPN	(Gift from Dr. Umetsu)	(PMab-1)	0.2 μg/ml
Moesin	Cell signaling	3150	1/150
FLAG*	Sigma-Aldrich	F3165	1/250
FLAG**	Cell signaling	2368	1/200
Nephrin	R&D	AF3159	1/300
pERM	Cell signaling	3726	1/400
Podocalyxin	R&D	MAB1556	1/100

### Electron microscopy

Kidneys were fixed by perfusion with 4% PFA and then immersed in 2.5% glutaraldehyde for 30 min. Subsequent preparation of SEM was performed using standard methods. We randomly selected 8–10 glomeruli in each mouse and 3 images at X8000 magnification were captured for evaluation of foot processes. The number of images containing foot processes that were more than twice as thick as normal ones were counted.

### Urinary platelets in mice with podocyte injury

As a podocyte injury model, we used NEP25 mouse line^41^, which expresses human (h) CD25 selectively in podocytes. Injection of an hCD25-targeted immunotoxin, LMB2, induces podocyte injury dose-dependently. In this study, NEP25 mice were injected with 5 ng/g body weight of LMB2. Five days after the LMB2 injection, urinary protein and blood were detected by Uropaper III UHAGKSpH 7S (Eiken), and then urine was collected and centrifuged at 500 g. The sediment was washed several times with PBS containing 0.1% BSA, EGTA 1 mM and PGE1 0.25 μM and stained with anti-CD41 antibody (Biolegend, #133,9011:100), and then inspected with confocal microscopy (ZEISS, LSM-880).

### Injection of DiI-labeled platelet solution into NEP25 mice

Platelets were isolated from the blood of C57BL/6 mice^42^ and stained with Vybrant DiI (Thermo Fisher scientific). A separate set of NEP25 mice were injected with LMB2. Five days later, DiI-labeled platelets were injected. The urine was collected and centrifuged at 180 g. Thereafter, the supernatant was centrifuged at 1300 g, according to the platelets isolation protocol^42^. The pellets were washed several times with washing buffer containing BSA, EGTA and PGE1, and then inspected with confocal microscopy (ZEISS, LSM-880).

### Statistical analyses

The results are expressed as the median and interquartile range (IQR). *P* values of < 0.05 were considered to indicate statistical significance. For Fig. 1B, C and F, differences between groups were analyzed using Paired sampled t-test. For Fig. 1E and 2D, ratio of Fc-CLEC-2/Fc or FLAG-GLEC-2/Control was obtained in each experiment, and the experiments were repeated 4 and 5 times, respectively. A one-sample t test was used to determine whether the mean ratio was different from 1. For other analyses, differences between groups were analyzed using the Mann–Whitney U‐test for continuous data. Statistical analyses were performed using the JMP software program (version 11, SAS Institute Inc.; Cary, NC, USA).

## Supplementary Information

Supplementary Figures.

## Data Availability

All data generated or analyzed during this study are included in this published article.
