# Supplementary material for: C-type lectin-like receptor (CLEC)-2, the ligand of podoplanin, induces morphological changes in podocytes
Source: Sci Rep. 2022 Dec 26;12:22356. doi: 10.1038/s41598-022-26456-9 (PMC9792514; doi:10.1038/s41598-022-26456-9)
Supplement: Supplementary file 1 — Supplementary Figures. [file 41598_2022_26456_MOESM1_ESM.pdf]

Supplementary Figure.1

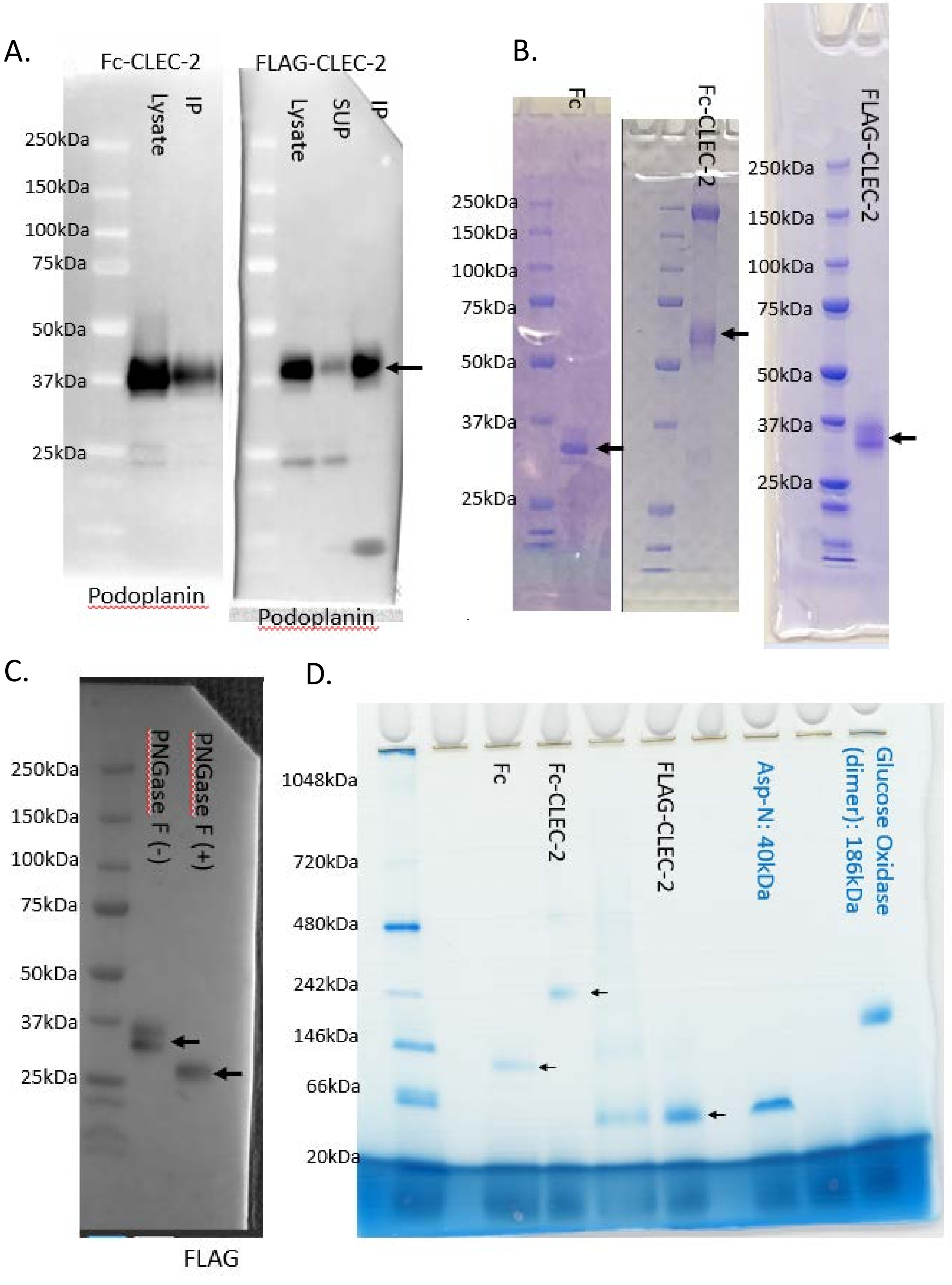

Supplementary Figure 1. Validation of recombinant CLEC-2 proteins

A) Pull-down assay. Fc-CLEC-2 and FLAG-CLEC-2 were incubated with podocyte lysate, then precipitated and assayed for Podoplanin (PDPN) by Western blot analysis. PDPN does not bind to agarose beads in the absence of CLEC-2 protein (reference 39). These data confirm that mouse PDPN can bind Fc-CLEC-2 and FLAG-CLEC-2. mouse PDPN: 36-45kDa. SUP: supernatant, IP: immunoprecipitation

B) SDS-PAGE and Coomassie Brilliant Blue (CBB) stain of recombinant proteins. The bands were detected as expected molecular size. Fc: 30kDa, Fc-CLEC-2: 60kDa, FLAG-CLEC-2: around 30-35kDa.

C) Deglycosylation assay of FLAG-CLEC-2 proteins. The deglycosylation by PNGase F changed the double bands (30/35 kDa) to a single band (26 kDa).

D) Blue Native PAGE of the recombinant proteins. Fc and Fc-CLEC-2 exist as tetramers (120kDa and 240kDa, respectively), and FLAG-CLEC-2 exists as a monomer (35kDa) in aqueous solution.

Supplementary Figure.2

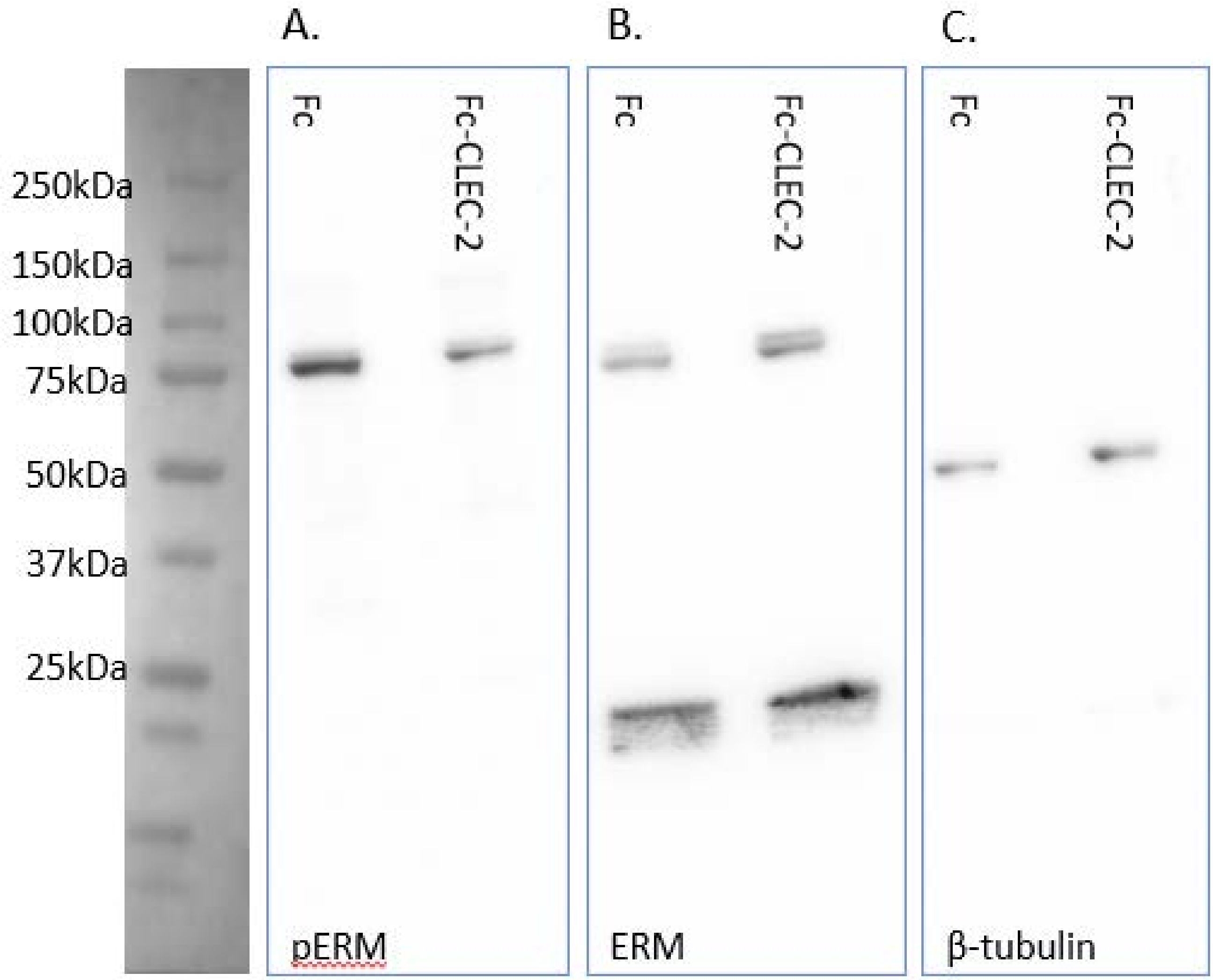

Supplementary Figure 2. Western blot analysis for ERM after treatment of Fc or Fc-CLEC-2 at 37°C for 1 hour.

A) The membrane was incubated with anti-pERM antibody
B) The same membrane was stripped off and incubated with anti-ERM antibody.
C) The same membrane was again stripped off and incubated with anti-β-tubulin antibody.

ERM proteins were dephosphorylated by Fc-CLEC-2, compared to Fc control.
Ezrin: 81kDa, Moesin: 75kDa, β-tubulin 55kDa.
Original images of the full-length membrane are shown in Supplementary Figure 3.

Supplementary Figure.3

A.

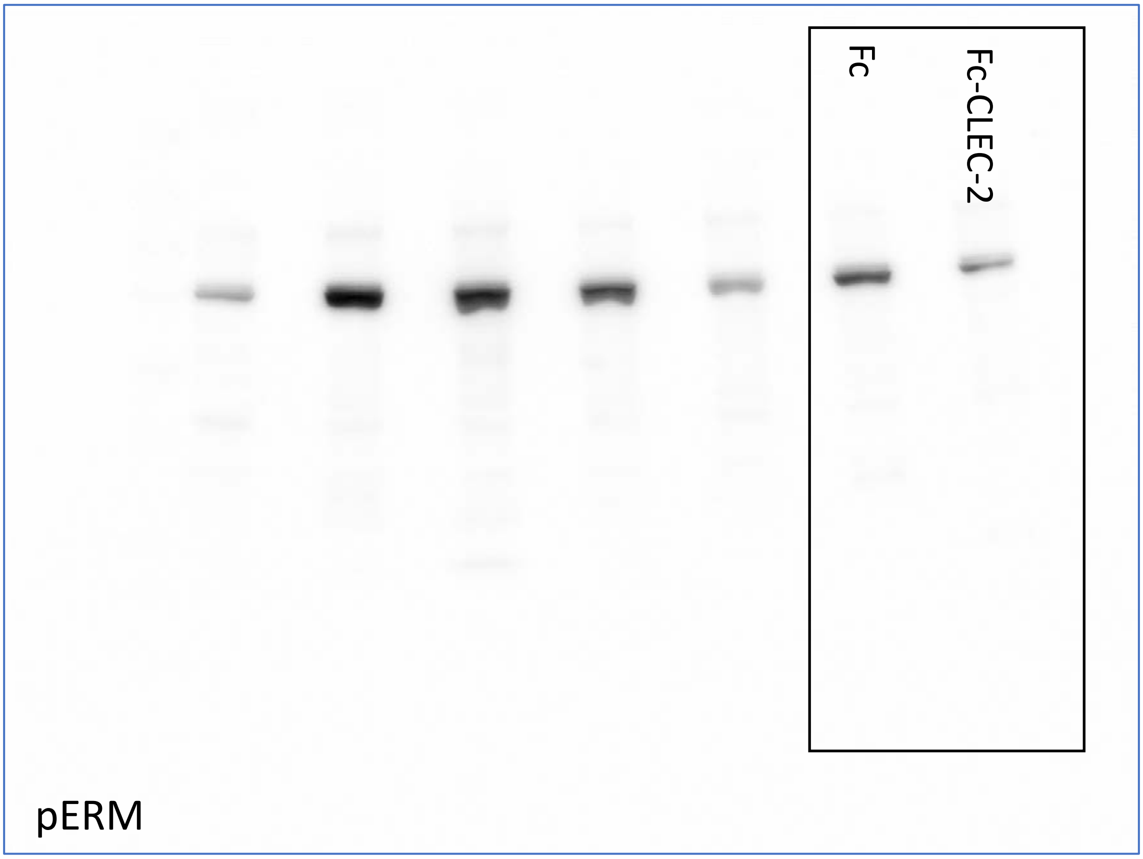

B.

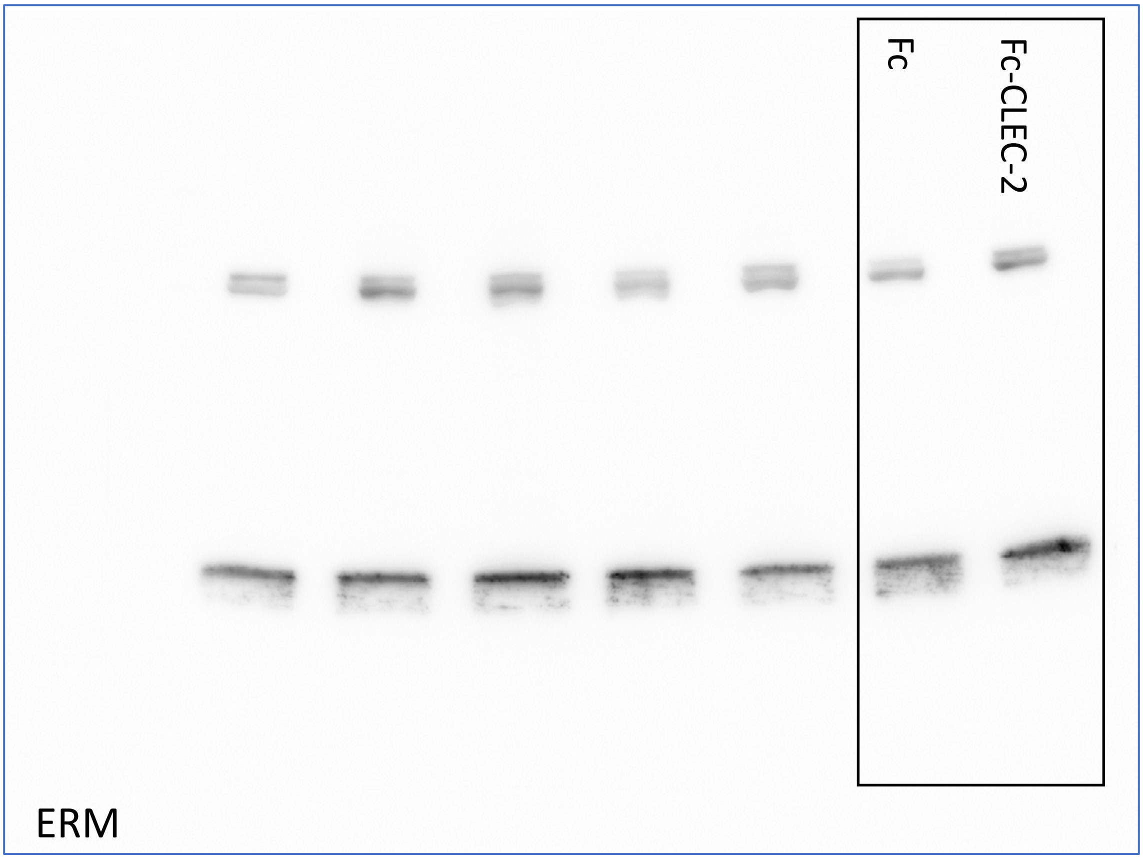

C.

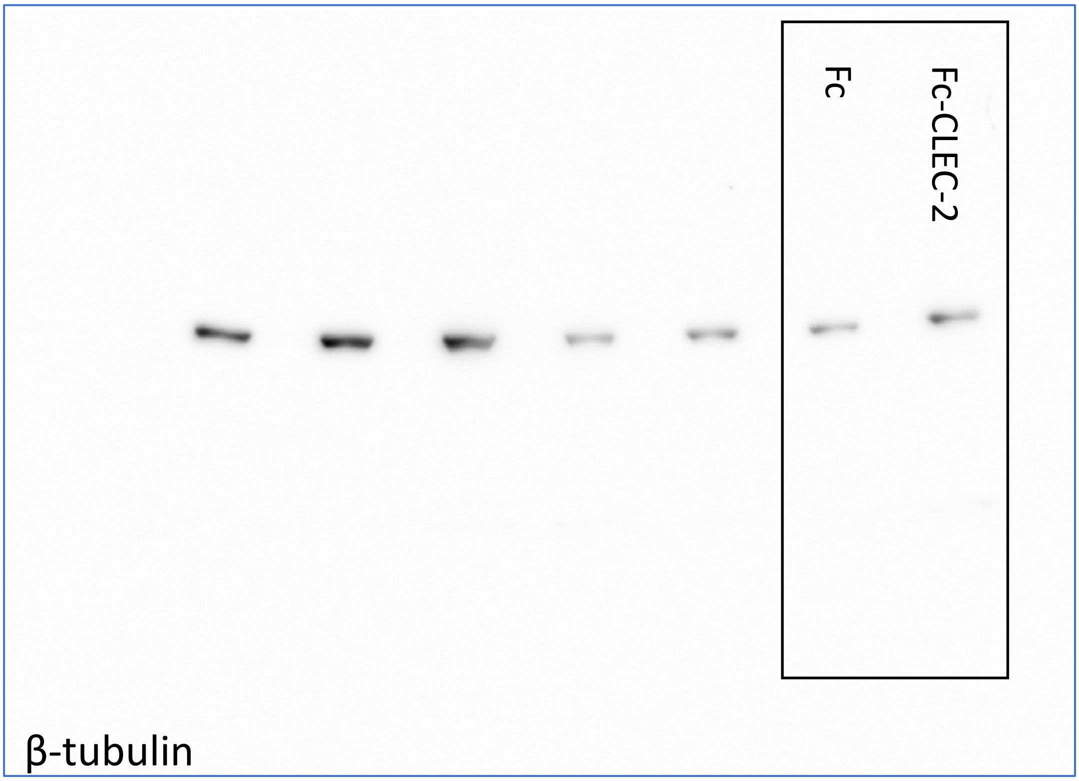

D.

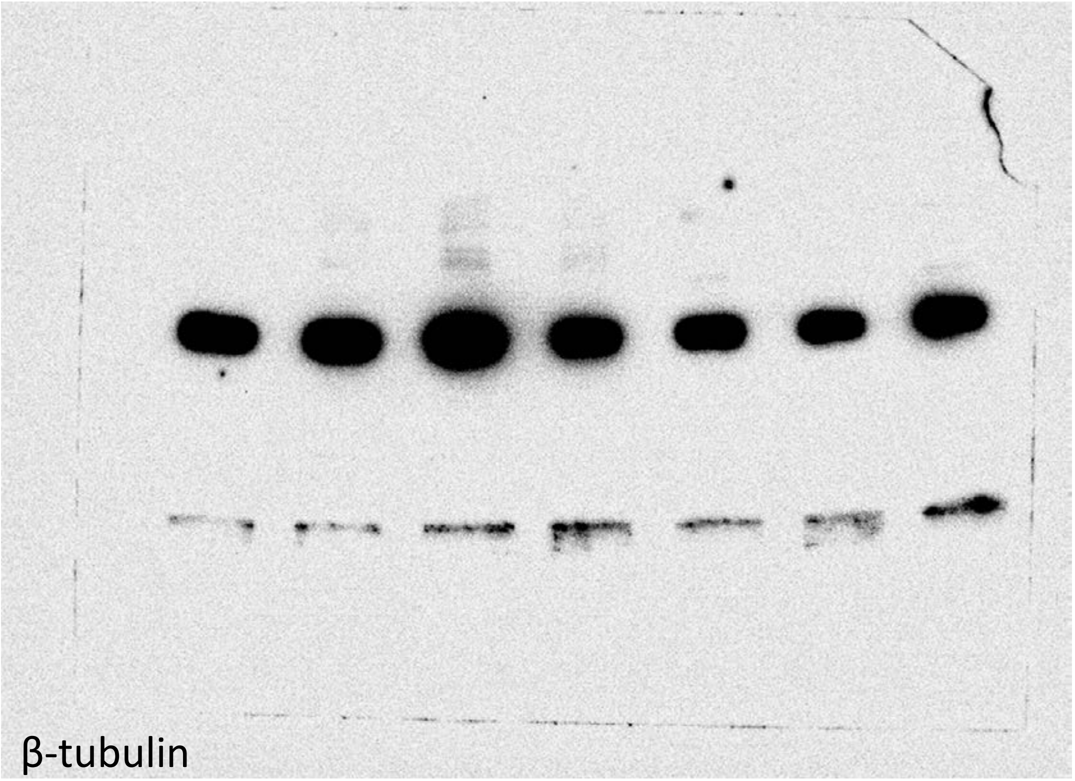

Supplementary Figure 3. Original images of Western blot analysis for ERM.
A) The original image for Supplementary Figure 2A.
B) The original image for Supplementary Figure 2B.
C) The original image for Supplementary Figure 2C.
D) High-contrast image of Supplementary Figure 3C to visualize the membrane edge.

Supplementary Figure.4

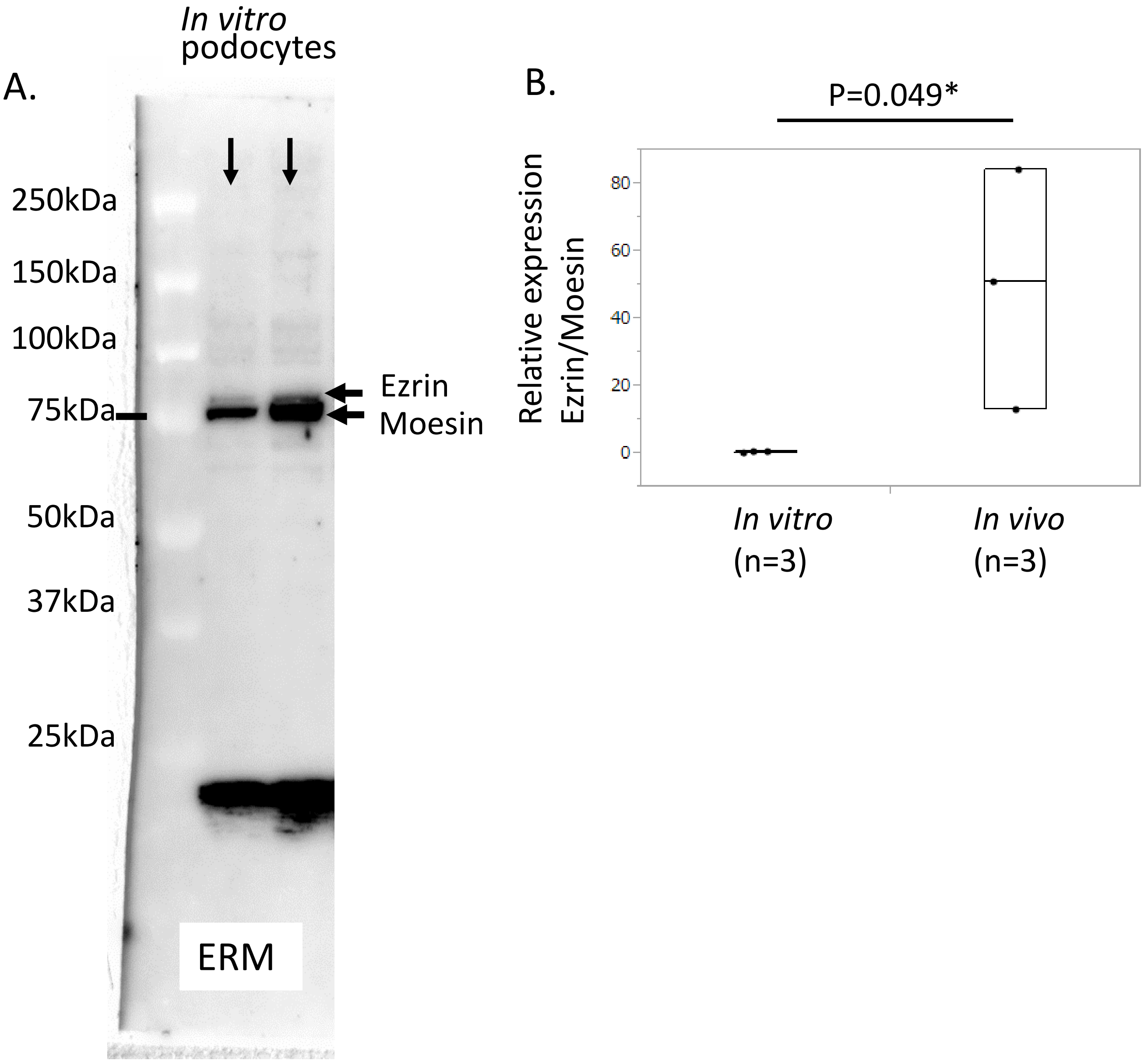

Supplementary Figure 4. ERM expression in cultured podocytes.

A) Western blot of lysate of cultured podocytes by anti-ERM antibody (middle and right lanes). Cultured podocytes contained more moesin (lower band, 75kDa) than ezrin (upper band, 81kDa). The left lane is a marker.

B) The relative mRNA expression of ezrin and moesin by quantitative PCR. Ezrin/Moesin ratio was 0.47(0.18-0.50) in cultured podocytes, while it was 51(13-84) in *in vivo* podocytes.

Supplementary Figure.5

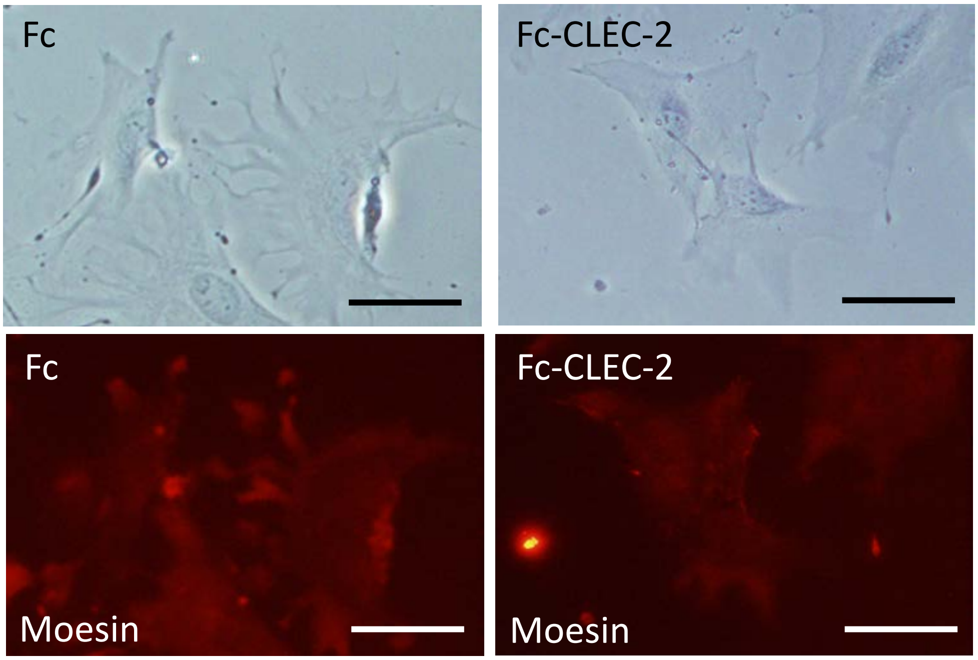

Supplementary Figure 5.
Phase contrast image and moesin staining of podocytes, after incubation with Fc or Fc-CLEC-2. Scale bar: 50μm

Supplementary Figure.6

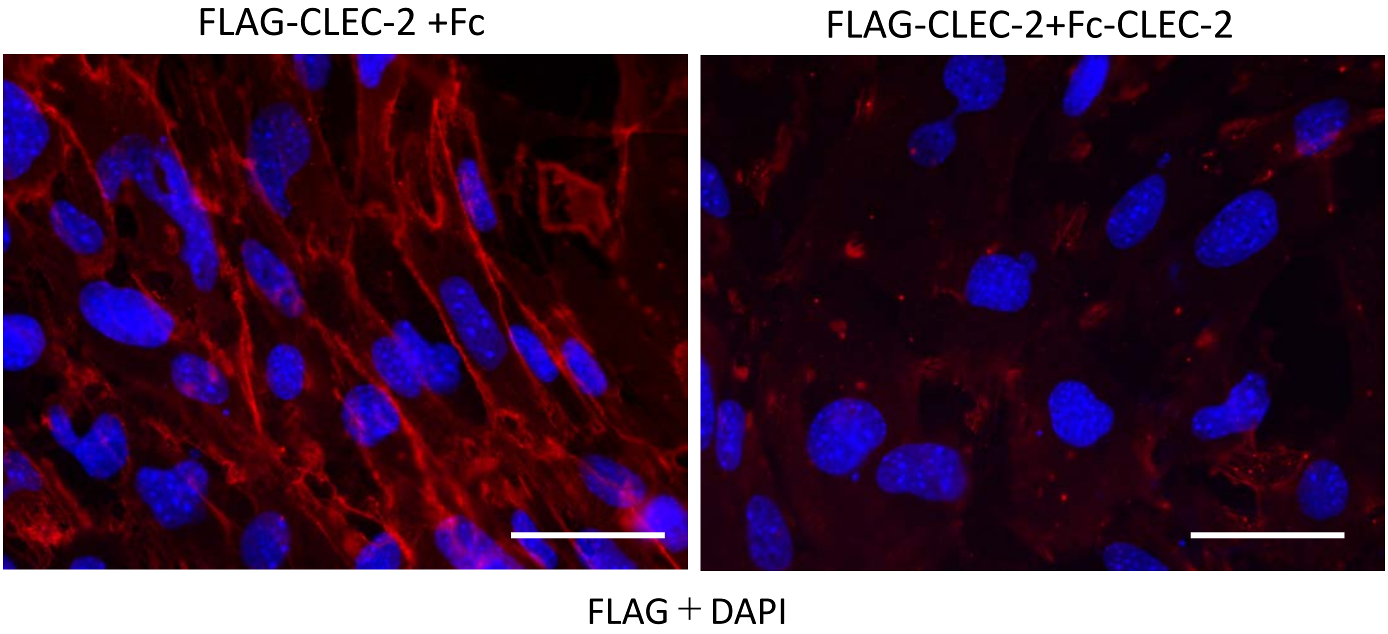

Supplementary Figure 6.
Immunostaining for FLAG of cultured podocytes, after the incubation with FLAG-CLEC-2 (10 μg/mL), in the presence of Fc (100 μg/mL) or Fc-CLEC-2 (200 μg/mL). The excess amount of Fc-CLEC-2, but not Fc, inhibited the binding of FLAG-CLEC-2 to podocytes, confirming the specificity of the binding of FLAG-CLEC-2. Measure bar: 50um

Supplementary Figure.7

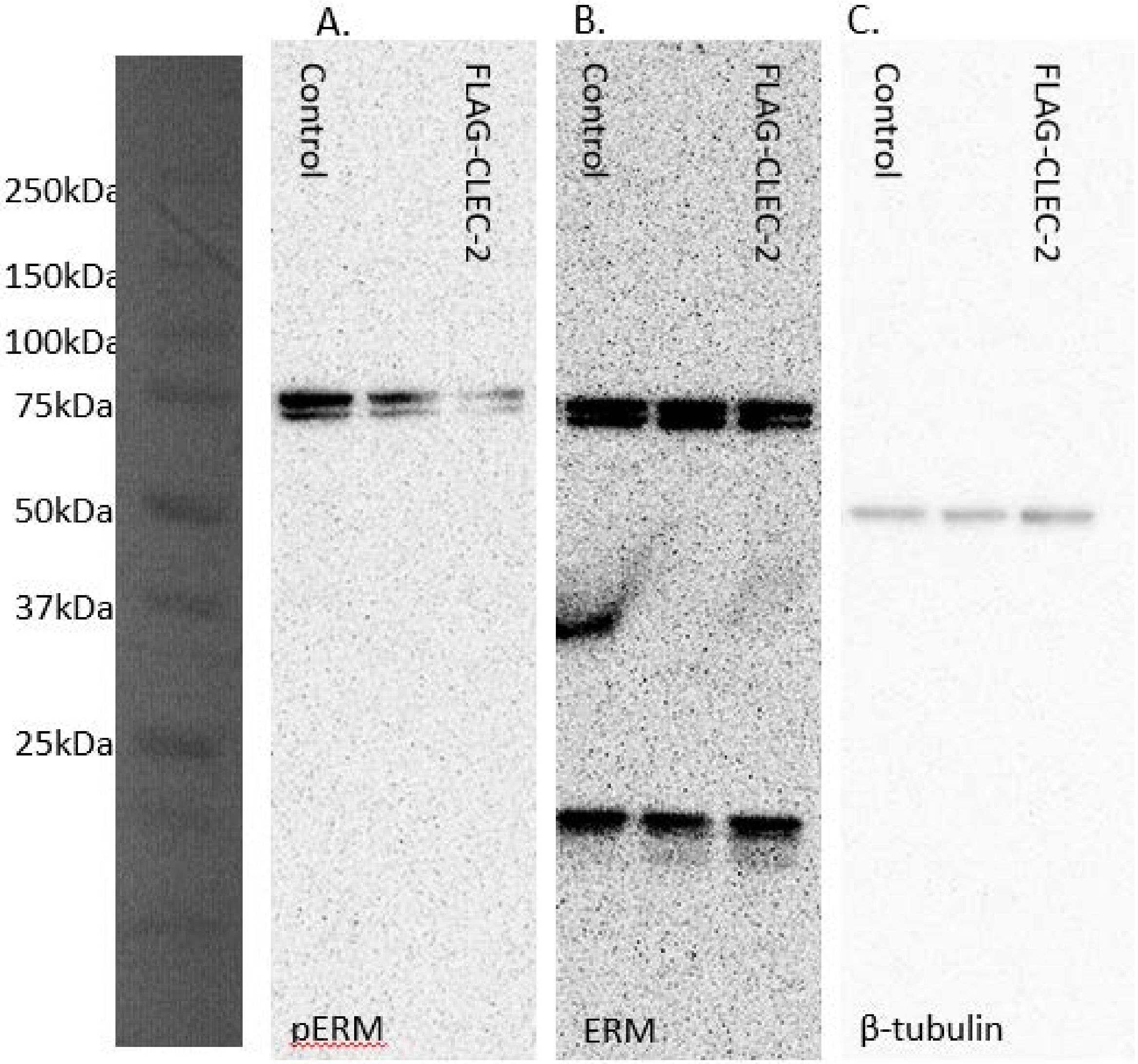

Supplementary Figure 7. Western blot analysis of glomerular lysate for ERM.

A) The membrane was incubated with anti-pERM antibody

B) The same membrane was stripped off and incubated with anti-ERM antibody.

C) The same membrane was again stripped off and incubated with anti-β-tubulin antibody.

ERM proteins were dephosphorylated by FLAG-CLEC-2.

Ezrin: 81kDa, Moesin: 75kDa, β-tubulin 55kDa.

Original images of each full-length membrane are shown in Supplementary Figure 8.

Supplementary Figure.8

A.

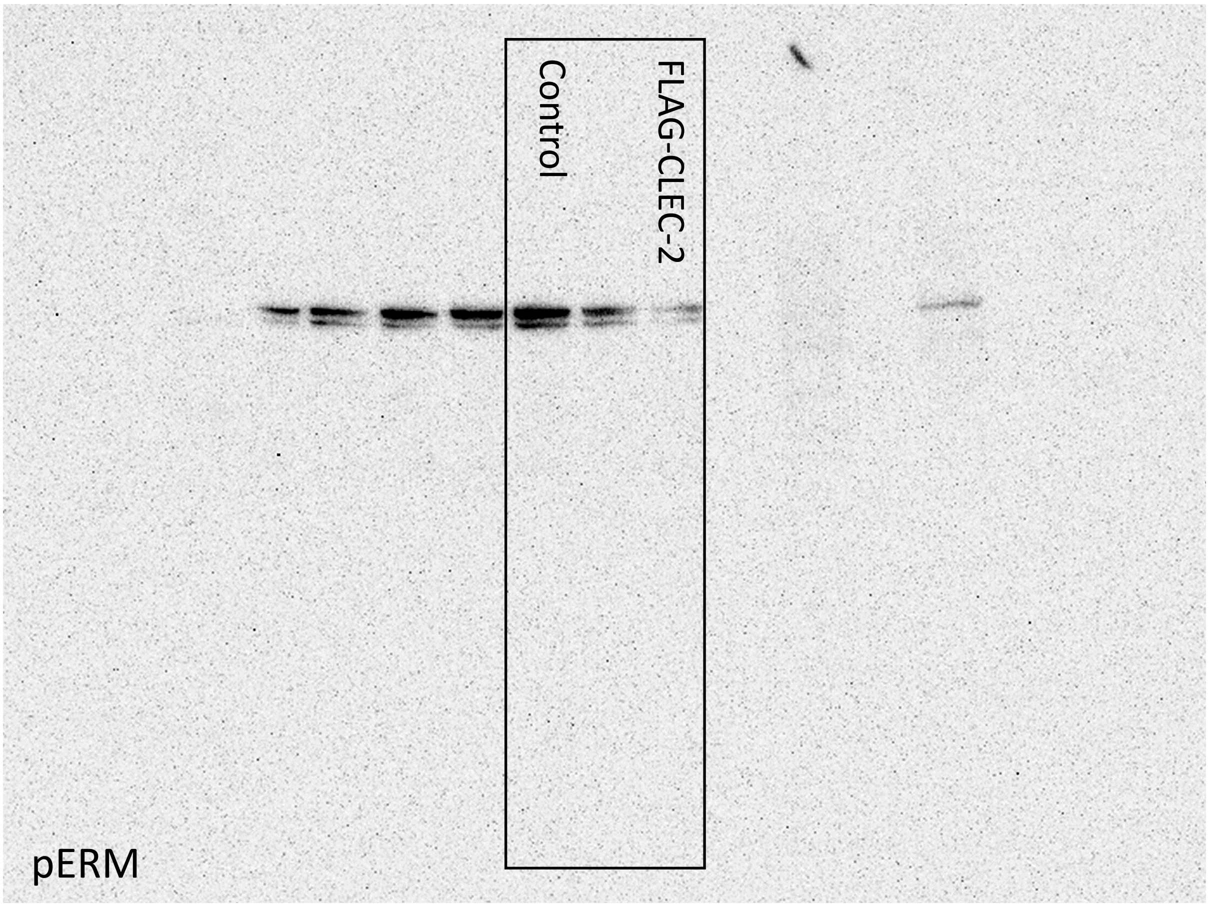

B.

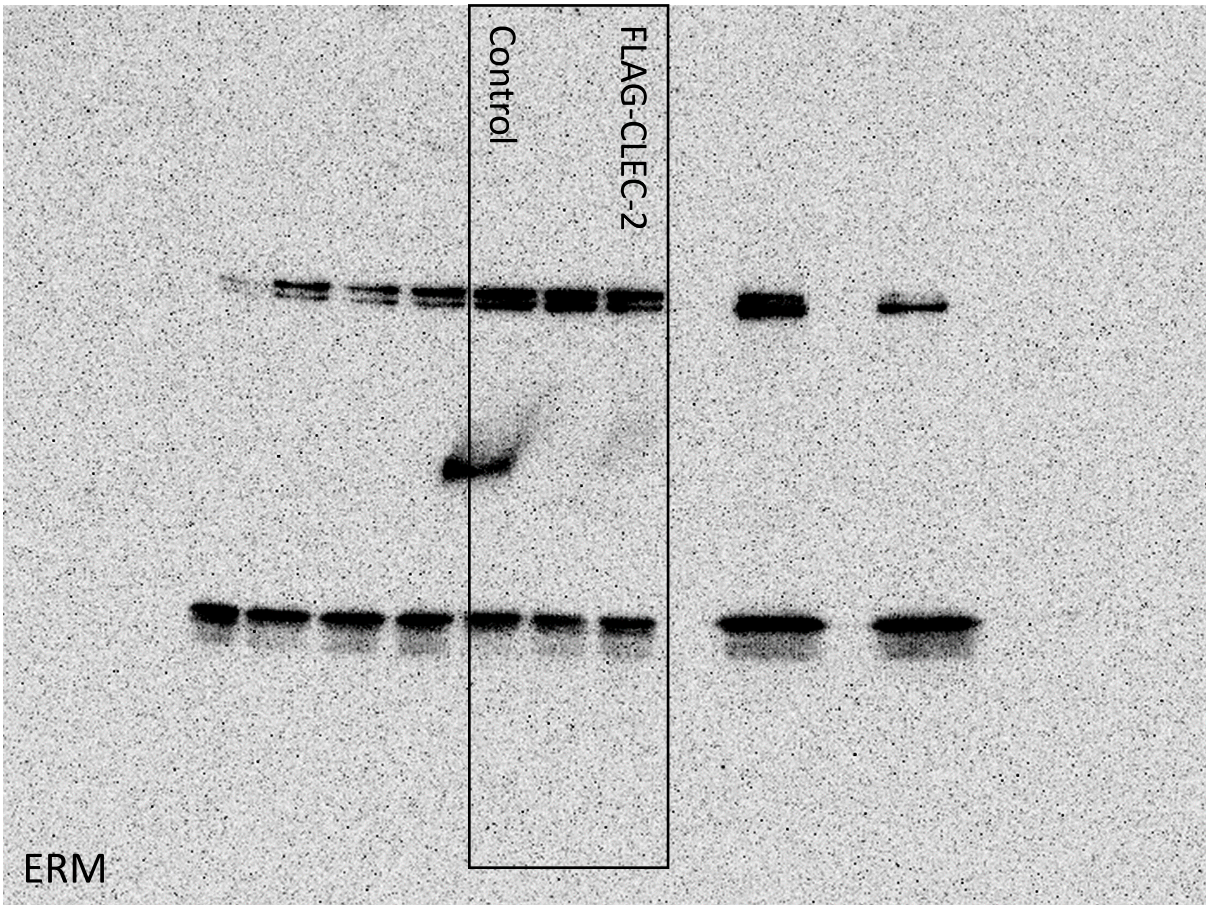

C.

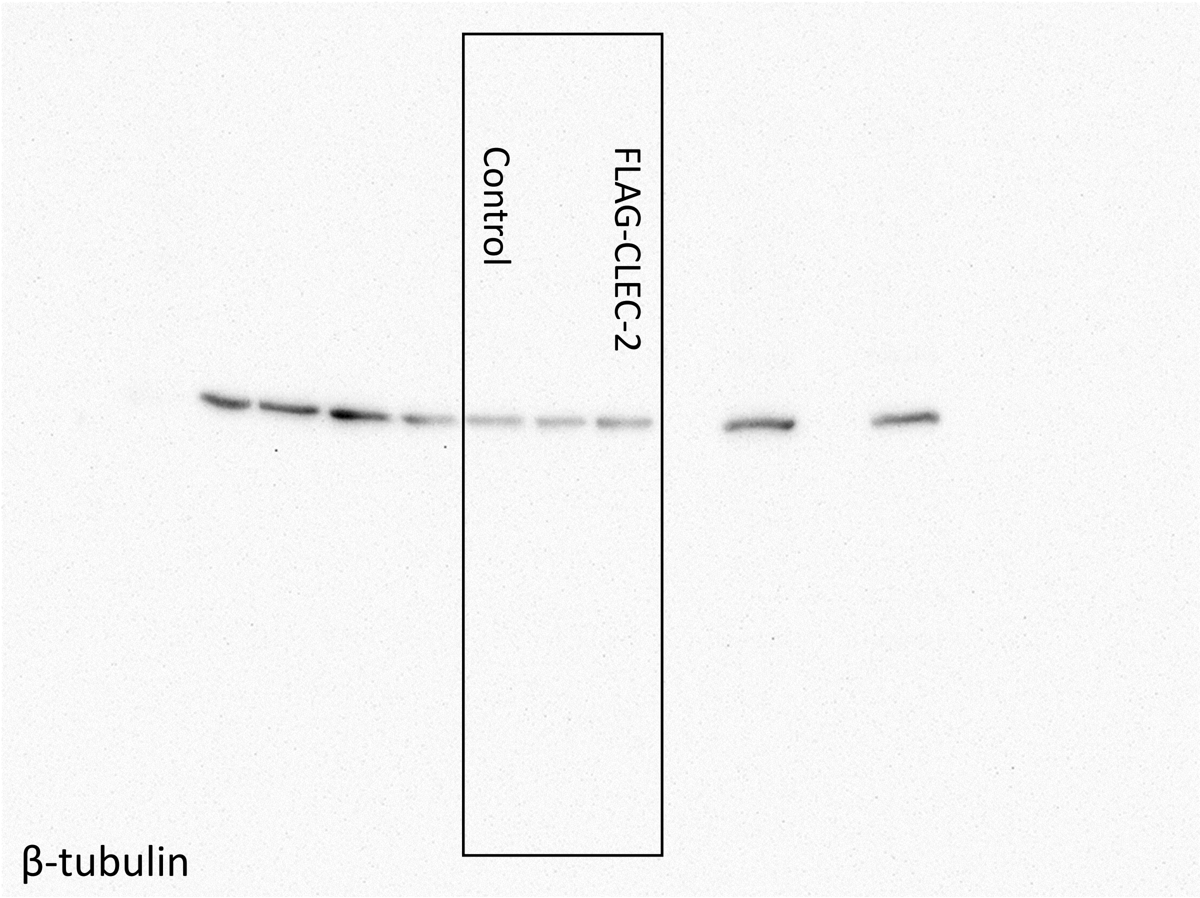

Supplementary Figure 8. Original images of Western blot analysis for ERM.
A) The original image for Supplementary Figure 7A.
B) The original image for Supplementary Figure 7B.
C) The original image for Supplementary Figure 7C.

Supplementary Figure.9

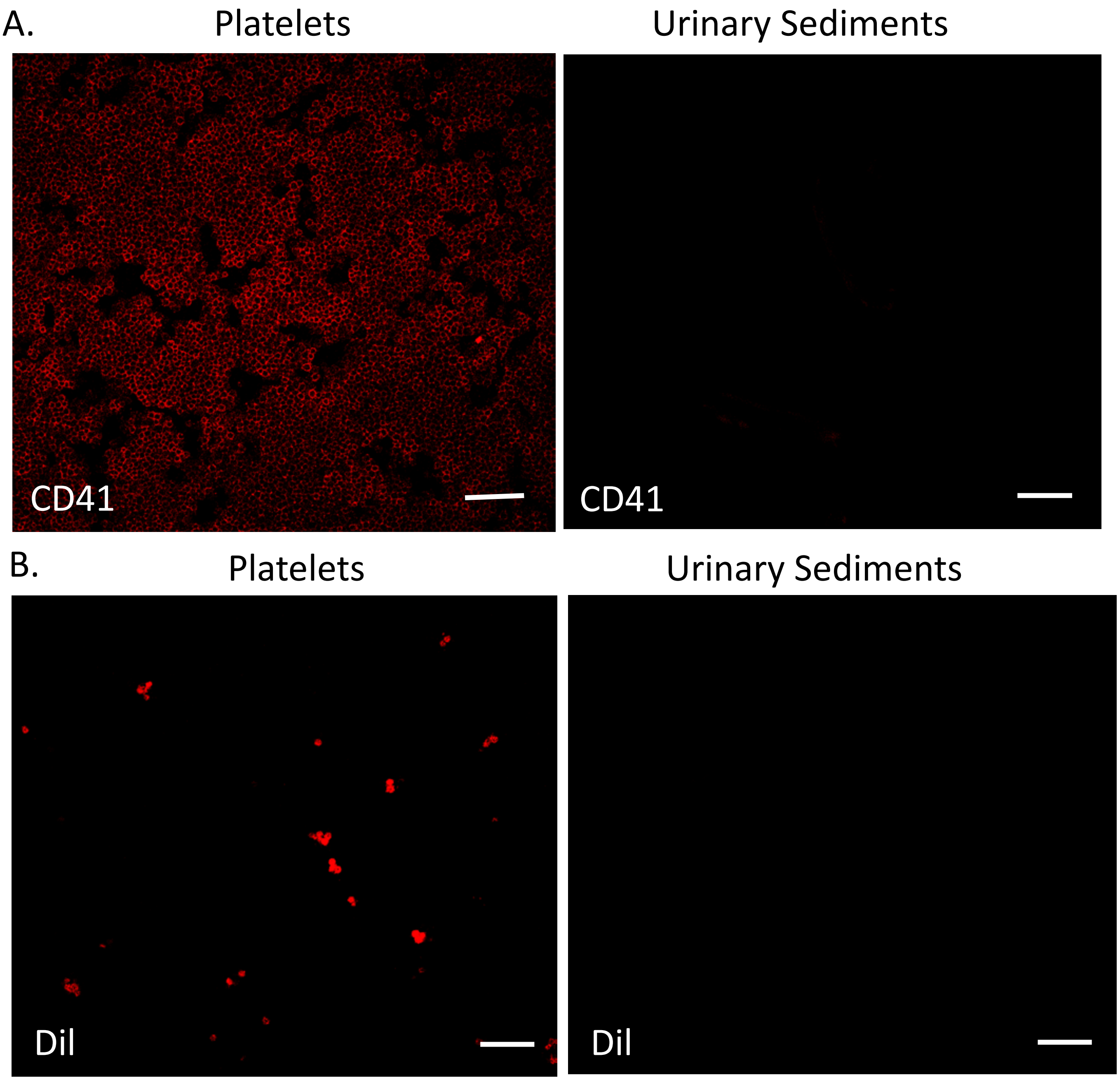

Supplementary Figure 9. Positive and negative control images for Figure 3B and 3C. Detection of platelets by confocal microscopy.

A) Left: Immunostaining for CD41 of platelets. Right: Urine sediment from normal mice immunostained for CD41.

B) Left: DiI labeled platelets. Right: Urine sediment from normal mice imaged by the same condition.

Scale Bar: 20μm.
